# Infection with SARS-CoV-2 following Second Dose Pfizer-BioNTech mRNA COVID-19 Vaccine BNT162b2 in Danish Adolescents Aged 12–18 Years: A Real-World Nationwide Danish Cohort Study

**DOI:** 10.3390/v16010056

**Published:** 2023-12-29

**Authors:** Nina Marie Birk, Anne Vinggaard Christensen, Ulrikka Nygaard, Henning Bundgaard, Susanne Dam Nielsen, Selina Kikkenborg Berg, Helle Wallach-Kildemoes

**Affiliations:** 1 Department of Cardiology, Copenhagen University Hospital Rigshospitalet, Inge Lehmanns Vej 7, 2100 Copenhagen, Denmark; 2 Department of Pediatrics and Adolescents, Herlev and Gentofte Hospital, Copenhagen University Hospital, 2730 Herlev, Denmark; 3 Faculty of Health and Medical Sciences, University of Copenhagen, Blegdamsvej 3B, 2200 Copenhagen, Denmark; ulrikka.nygaard@regionh.dk; 4 Department of Pediatrics and Adolescent Medicine, Copenhagen University Hospital Rigshospitalet, Blegdamsvej 9, 2100 Copenhagen, Denmark; 5 Department of Infectious Diseases, Copenhagen University Hospital Rigshospitalet, Blegdamsvej 9, 2100 Copenhagen, Denmark; susanne.dam.poulsen@regionh.dk; 6 Department of Clinical Medicine, University of Copenhagen, 2200 Copenhagen, Denmark

**Keywords:** COVID-19, BNT162b2 vaccine, vaccine effectiveness, adolescents, real-world data

## Abstract

In this real-world cohort study based on Danish nationwide registers, the cumulated proportion, relative risk (RR) of SARS-CoV-2 breakthrough infections, and vaccine effectiveness (VE) were investigated in adolescents aged 12–18 years following vaccination with the BNT162b2 vaccine compared to unvaccinated controls. Adolescents with and without vaccination with the first dose of BNT162b2 between 1 May and 30 September 2021 were included. Effect estimates include proportions with a positive SARS-CoV-2 RT-PCR test among vaccinated and unvaccinated, RR, and VE at three different time points. During Delta-dominance, VE was first 97.6% (95% CI 96.3–98.4), then 96.2% (95% CI 95.4–96.9) in the age group 12–15 and 95.1% (95% CI 94.1–96.0) followed by 95.5% (95% CI 94.8–96.1) in the age group 16–18 years, respectively. During Omicron dominance, VE was 5.8% (95% CI 4.6–7.0) in ages 12–15 years and 9.2% (95% CI 7.7–10.6) in ages 16–18 years. Thus, BNT162b2-vaccine protection was limited during the Omicron era.

## 1. Introduction

Knowledge of SARS-CoV-2 transmission following COVID-19 vaccination in adolescents is pivotal from a public health perspective for defining and evaluating transmission mitigation strategies. From an immunological perspective, this topic is important to improve insight into immune evasion by specific circulating viral variants.

Adolescents play a key role in terms of SARS-CoV-2 transmission [1], and effective vaccination within this group is, therefore, important in disease prevention and reaching herd immunity. Furthermore, to minimize the pandemic’s educational and social disruption [2], knowledge of vaccine effectiveness in adolescents is paramount.

In Denmark, adolescents were invited for COVID-19 vaccination with the BNT162b2 vaccine (Pfizer-BioNTech) from May 2021 (age 16–18 years) and July 2021 (age 12–15 years) [3]. Due to spike protein mutations, new variants of SARS-CoV-2 continuously emerge, causing a potential challenge to vaccine protection [4]. The B.1.1.529 Omicron variant of SARS-CoV-2 became dominant in Denmark in December 2021, followed by a rapid increase in infection rates.

In a real-world cohort consisting of SARS-CoV-2 uninfected adolescents, we aimed to explore the difference in infection rates between vaccinated and unvaccinated adolescents during the transition of SARS-CoV-2 variant dominance from Delta to Omicron. Thus, we investigated the cumulated proportion, relative risk (RR) of SARS-CoV-2 breakthrough infections, and vaccine effectiveness (VE) in adolescents aged 12–18 years following vaccination with the COVID-19 BNT162b2 vaccine, compared to unvaccinated controls.

## 2. Materials and Methods

This real-world cohort study was based on Danish nationwide registers, accessed through a secure platform through the Danish Health Data Authority. From the Danish Vaccination Register [5], date and type of vaccination against SARS-CoV-2 was obtained. Date(s) of positive test(s) RT-PCR SARS-CoV-2 test were obtained from the Danish Microbiology Database [6]. Data from the national registers were linked at the individual level using an encrypted version of the unique Danish Civil Registration Number issued to all Danish citizens at birth or data from work and/or residence permits.

The study population covered Danish adolescents aged 12–18 years with no prior positive SARS-CoV-2 RT-PCR test sampled from the LongCOVIDKidsDK study [7]. In the present study, adolescents with and without vaccination with the first dose of BNT162b2 between 1 May and 30 September 2021 were included.

To account for seasonal variations in healthcare access, rollout of vaccination programs, and SARS-CoV-2 infection load, each week during the inclusion period (following the age-specific vaccine rollout), we randomly sex and age group matched each first-dose vaccination during the actual sampling week to one unvaccinated cohort member. Any cohort member of the same sex and age group not vaccinated or who previously tested SARS-CoV-2 positive by the end of the actual sampling week was eligible as an unvaccinated reference for a first-dose vaccinated adolescent.

Applying sampling by replacement [8], each first-dose vaccinated adolescent was randomly matched 1:1 by sex and age group (12–15 and 16–18 years of age) with an unvaccinated peer each week during the inclusion period. Hence, each unvaccinated individual could be matched to several vaccinated.

The second-dose vaccine population was sampled from the first-dose vaccine population, applying an analogous weekly matching and sampling strategy, excluding individuals with a positive SARS-CoV-2 test prior to the actual sampling week.

The second-dose vaccine population was followed in the registries for their first positive SARS-CoV-2 RT-PCR test, applying three follow-up periods after the index date: short (0–21 days), medium (0–56 days), and long (57–182 days). While the index date for vaccinated adolescents corresponded to the second dose vaccination date, the index dates for the unvaccinated peers were assigned corresponding to the Wednesday of the sampling week. Unvaccinated peers were only included within the specific follow-up period, provided they were not first-dose vaccinated between the index date and the end of follow-up.

Effect estimates are given as proportions of adolescents with a positive SARS-CoV-2 RT-PCR test in the vaccinated and unvaccinated group and as RR and VE at three different time points. While RR was calculated as the risk of being SARS-CoV-2 infected among vaccinated divided by the risk among unvaccinated, VE was calculated as (1 − RR) × 100 [9]. 

## 3. Results

The study population consisted of 103,194 fully vaccinated and 24,055 unvaccinated adolescents. As some of the unvaccinated adolescents had the first dose of vaccine after the index date, a decreasing number of unvaccinated adolescents were followed during the follow-up period. Conversely, with a longer follow-up period, an increasing number of adolescents were fully vaccinated (Table 1).

During the short follow-up, cumulated proportions of SARS-CoV-2 infection were 2.0% vs. 0.1% (unvaccinated vs. vaccinated) in the 12–15-year-olds and 4.6% vs. 0.2% among the 16–18-year-olds. During medium follow-up, cumulated proportions were 5.8% vs. 0.2% (unvaccinated vs. vaccinated) in the 12–15-year-olds and 8.5% vs. 0.4% among the 16–18-year-olds. During long follow-up, the proportion of infected individuals increased to 78.8% vs. 74.3% (unvaccinated vs. vaccinated) in the 12–15-year-olds and 74.8% vs. 67.9% among the 16–18-year-olds (Table 1).

During the short follow-up, the RR was 0.02 (95% CI 0.02–0.04) for getting a positive SARS-CoV-2 RT-PCR test for vaccinated vs. unvaccinated 12–15-year-olds. The 16–18-year-olds had an RR of 0.05 (95% CI 0.04–0.06) during the same time period. During the medium follow-up, the RR of SARS-CoV-2 infection was 0.04 (95% CI 0.03–0.05) for the 12–15-year-old adolescents and 0.05 (95% CI 0.04–0.05) for the 16–18-year-old adolescents. During the long follow-up, the RR increased to 0.94 (0.93–0.95) in the 12–15-year-olds and 0.90 (0.89–0.92) in the 16–18-year-old adolescents. At all time points, the unvaccinated group had a significantly higher relative risk of SARS-CoV-2 infection compared to the vaccinated adolescents (Table 1).

VE was 97.6% (95% CI 96.3–98.4) in the 12–15-year-olds and 95.1 (95% CI 94.1–96.0) in 16–18-year-olds during short follow-up. During the medium follow-up, VE was 96.2% (95% CI 95.4–96.9) for the 12–15-year-old adolescents and 95.5% (95% CI 94.8–96.1) for the 16–18-year-old adolescents. VE decreased to 5.8% (95% CI 4.6–7.0) in the 12–15-year-olds and 9.2% (95% CI 7.7–10.6) in the 16–18-year-old adolescents.

**Figure 1:**
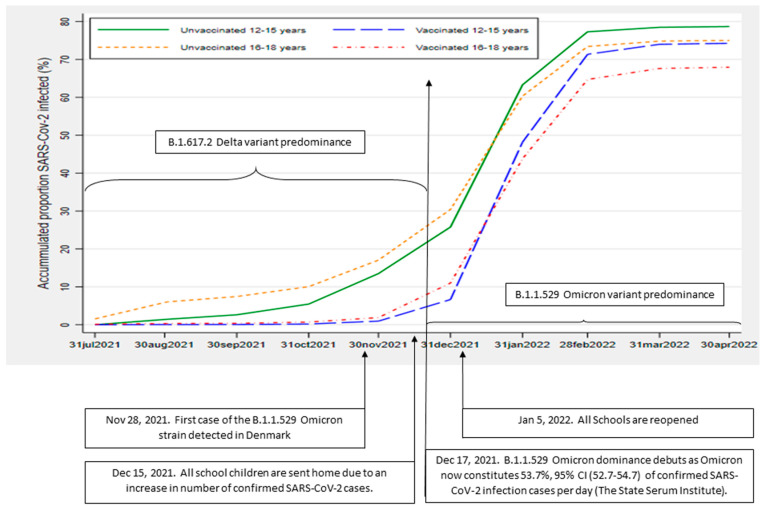
Accumulated proportions of SARS-CoV-2 infected adolescents over time, according to vaccination status and age group. Depicting the accumulated proportion (%) of SARS-CoV-2 RT-PCR test-positive Danish adolescents in the age groups 12–15 and 16–18 years for both COVID-19 vaccinated and unvaccinated individuals in the chosen timespan of 31 July 2021 to 30 April 2022. Vaccination was defined as having received the recommended two doses of Pfizer-BioNTech mRNA COVID-19 vaccine BNT162b2. Inclusion of uninfected adolescents—vaccinated or unvaccinated—between May 1 and 30 September 2021.

Before 28 November 2021, when the Omicron strain entered Denmark, less than two percent of vaccinated adolescents had been infected. Thereafter, however, we observed an acceleration in the proportion of breakthrough infections (Figure 1). The curves representing the cumulated proportion of SARS-CoV-2 infection were almost parallel and showed a marked increase across vaccination groups in both age groups from the end of December 2021. This coincides with Omicron variant dominance (17 December 2021) and the reopening of the schools (5 January 2022) (Figure 1).

## 4. Discussion

The study showed that cumulated proportions of SARS-CoV-2 infections were low in NT162b2 vaccinated adolescents during Delta variant dominance; however, they markedly increased and approached similar levels as in unvaccinated adolescents during Omicron variant dominance. Our findings demonstrate the marked reduction in vaccine effectiveness from a VE of 95.1–97.6% during Delta dominance to a VE of 5.8–9.2% during Omicron dominance.

In line with this study, a previous study found a significantly lower BNT162b2 VE in adolescents aged 12–15 years against Omicron compared to the Delta variant (59% vs. 87%) during a follow-up period of 14–149 days [10]. The longer follow-up period of 182 days may explain some of the reduced effectiveness against Omicron found in our study, as approximately 75% of vaccinated individuals aged 12–15 years were infected at the end of follow-up, where Omicron constituted more than 90% of daily cases in Denmark. In the present study, we were not able to distinguish between symptomatic and asymptomatic infections, as in the study reported above [10]. Several studies showed the effects of a third booster dose on VE with regards to both SARS-CoV-2 infection and COVID-19 disease in the age span of 12–15 years old [11], 12–17 years [12,13], and 12–18 years [14]. It was not within the framework of this study to assess the effect of one or more BNT162b2 booster doses.

An important strength of this study is the use of real-world data from the high-quality Danish nationwide administrative registries, enabling us to both link and follow this cohort of Danish adolescents in the age of 12–18 years. The complete and updated individual-level information on COVID-19 vaccination type and status, as well as SARS-CoV-2 RT-PCR test results, strengthen the validity of our results. Further, the weekly sampling and matching strategy represents another strength, taking the rapidly changing SARS-CoV-2 load and continuous vaccination rollout into account. A limitation of this study is a lack of data on rapid on-site self-collected nasal swab tests and a lack of data on negative test results. Denmark did have an intensive COVID-19 test strategy with mandatory school tests on days 4 and 6 for both vaccinated and unvaccinated adolescents following close contact with a test-positive person and confirmational SARS-CoV-2 RT-PCR test in case of a positive rapid test. This strengthens the assumption that the lack of a positive SARS-CoV-2 RT-PCR test truly represents a COVID-19 naïve adolescent. Another limitation is the lack of information on symptoms and COVID-19 disease, and, therefore, we cannot conclude the vaccine’s effect on symptomatic versus asymptomatic disease. Additionally, since we had no mandatory tests, we may have missed some of the asymptomatic infections.

In this real-world nationwide Danish cohort study, the cumulated proportion of SARS-CoV-2-infected vaccinated adolescents was low and slowly progressing, and the VE was high in the context of B.1.617.2 Delta variant predominance. However, in the context of Omicron variant dominance, an acceleration in breakthrough infections, as well as infections in the non-vaccinated adolescents, was observed. Further, VE was almost repealed, decreasing by 90.4% in the 12–15-year-olds and 86.3% in the 16–18-year-olds, respectively. Vaccinated adolescents, however, remained significantly less infected compared to controls throughout follow-up across both age groups. At the end of follow-up, the cumulated proportions of infected adolescents—both vaccinated and unvaccinated—were high and increasingly similar, reaching levels of a minimum of 67.9%. Future studies may illuminate at which possible time point the difference between vaccination groups ceases to be significant.

Interestingly, the 12–15-year-old adolescents, both vaccinated and unvaccinated, reached higher proportions of infection at the end of follow-up compared to the 16–18-year-old adolescents. This may lead to the thought that Omicron was most immune evasive within this age group. However, since vaccinated and unvaccinated adolescents in both age groups had parallel curves of cumulated proportions of infection (Figure 1), the explanation is most likely due to differences in behavior (less adherence to the guidelines and more social activities in the 12–15-year-olds). Therefore, this may result in a different level of exposure to infection between the two age groups. This observation may be important to pursue. Both age groups were fully vaccinated well before Omicron’s entrance; hence, the difference in time of vaccination in relation to the time of the Delta–Omicron dominance shift is not likely to explain the findings.

## 5. Conclusions

In conclusion, this Danish nationwide real-world cohort study confirmed that two doses of the BNT162b2 vaccine effectively protected adolescents from SARS-CoV-2 infection in times of Delta variant dominance. However, during Omicron variant dominance, we found a marked reduction in VE and, consequently, rapid infection spread.

## Figures and Tables

**Table 1:** Risk of getting SARS-CoV-2 infection among adolescents in Denmark according to vaccination status, age group, and follow-up periods.

Follow-Up Period ^1^	12–15-Year-Olds									16–18-Year-Olds				
	Unvaccinated ^2^		Vaccinated ^3^		Relative Risk (95% CI) ^5^			Unvaccinated ^2^		Vaccinated ^3^		Relative Risk (95% CI) ^5^		
	N	Proportion SARS-CoV-2 Infection (%) ^4^	N	Proportion SARS-CoV-2 Infection (%) ^4^		Vaccine Effectiveness (VE) (%) (95% CI) ^6^	*p* Value ^6^ (VE)	N	Proportion ^4^ SARS-CoV-2 Infection (%)	N	Proportion ^4^ SARS-CoV-2 Infection (%)		Vaccine Effectiveness (VE) (%) (95% CI) ^6^	*p*-Value ^6^ (VE)
Short	14,543	2.0	45,893	0.1	0.02 (0.02–0.04)	97.6 (96.3–98.4)	<0.001	9512	4.6	57,301	0.2	0.05 (0.04–0.06)	95.1 (94.1–96.0)	<0.001
Medium	13,451	5.8	46,720	0.2	0.04 (0.03–0.05)	96.2 (95.4–96.9)	<0.001	8220	8.5	58,394	0.4	0.05 (0.04–0.05)	95.5 (94.8–96.1)	<0.001
Long	8158	78.8	46,733	74.3	0.94 (0.93–0.95)	5.8 (4.6–7.0)	<0.001	5505	74.8	58,583	67.9	0.90 (0.89–0.92)	9.2 (7.7–10.6)	<0.001

^1^. Follow-up periods after index date: Days after index date: Short = 0–21 days; Medium = 0–56 days; Long: 57–182 days (excluding individuals who tested SARS-CoV-2 infection positive before day 57). Observations until May 2022. ^2^. Unvaccinated are adolescents unvaccinated throughout the follow-up period. ^3^. Vaccinated are adolescents vaccinated with 2 doses of COVID-19 vaccine = index date. ^4^. Proportion tested positive for SARS-CoV-2 infection during follow-up. ^5^. Relative risk (RR): Risk of SARS-CoV-2 infection among vaccinated relative to unvaccinated. In parenthesis: 95% relative risk confidence interval. ^6^. Vaccine effectiveness (VE) is calculated as (1 − RR) × 100.

## Data Availability

Deidentified individual participant data will not be made available.
